# Virulence and Antimicrobial Resistance Pattern of *Aeromonas* spp. Colonizing European Pond Turtles *Emys orbicularis* and Their Natural Environment. First Study from Poland

**DOI:** 10.3390/ani11102772

**Published:** 2021-09-23

**Authors:** Leszek Guz, Aneta Nowakiewicz, Krzysztof Puk, Przemysław Zięba, Sebastian Gnat, Łukasz Matuszewski

**Affiliations:** 1 Sub-Department of Biology and Fish Diseases, Department of Parasitology and Fish Diseases, Faculty of Veterinary Medicine, University of Life Sciences, Akademicka 12, 20-033 Lublin, Poland; leszek.guz@up.lublin.pl (L.G.); krzysztof.puk@up.lublin.pl (K.P.); 2 Sub-Department of Veterinary Microbiology, Department of Preclinical Veterinary Sciences, Faculty of Veterinary Medicine, University of Life Sciences, Akademicka 12, 20-033 Lublin, Poland; sebastian.gnat@up.lublin.pl; 3 State Veterinary Laboratory, Droga Męczenników Majdanka 50, 20-325 Lublin, Poland; przemekzieba@gmail.com; 4 Department of Pediatric Orthopedics and Rehabilitation, Faculty of Medicine, Medical University, Gębali 6, 20-093 Lublin, Poland; lukasz.matuszewski@umlub.pl

**Keywords:** *Aeromonas*, antimicrobial resistance, *Emys orbicularis*, MALDI-TOF-MS, virulence, wildlife

## Abstract

**Simple Summary:**

*Aeromonas* species are Gram-negative facultative anaerobic bacteria with a wide distribution in nature. The genus comprises bacteria which can cause different types of diseases in many warm- and cold-blooded animals. Moreover, exposure to these bacteria can cause gastroenteritis or localized skin infections in humans. In the current study, *Aeromonas* species were identified in samples from water sources and European pond turtles (*Emys orbicularis*) from Eastern Poland. Our results revealed a high prevalence of *Aeromonas* isolates (43.2%) in the turtles and 66.7% in the water samples. All isolates were resistant to ampicillin, 62% to sulfamethoxazole, 40.5% to erythromycin, and 40.5% to colistin. Eight strains were intermediately resistant to meropenem. Additionally, most *Aeromonas* isolates (from 90.5% to 45.2%) were found to possess the following virulence genes: *fla, aer, hly*A, *act, ela, alt*, and *ast*. This report indicated that European pond turtles were colonized by *Aeromonas* spp. While acting as reservoirs for these pathogens, they may play an important role in the etiology of *Aeromonas*-associated animal infections. Moreover, the undesirable properties of water quality caused by the presence of drug-resistant aeromonads could exert a negative impact on human health.

**Abstract:**

The aim of the study was to isolate and identify species belonging to the *Aeromonas* genus and evaluate the antimicrobial resistance and virulence patterns of isolates colonizing European pond turtles (*Emys orbicularis*) from natural environment of Eastern Poland. In total, 74 turtles and 15 samples of water from their natural environment were examined. More than 40 strains were isolated and identified: *A. bestiarum* (*n* = 1), *A. hydrophila* (*n* = 13), *A. allosaccharophila* (*n* = 2), *A. salmonicida* (*n* = 3), and *A. veronii* (*n* = 23). The highest incidence of resistance was noted for ampicillin (100%) and sulfamethoxazole (62.0%), followed by erythromycin and colistin (both 40.5%). Moreover, eight strains were intermediately resistant to meropenem (19%). Most *Aeromonas* isolates were found to possess more than one virulence gene among *fla, aer, hly*A, *act, ela, alt*, and *ast*. We showed that the population of free-living European pond turtles was highly colonized by *Aeromonas* spp. Such strains may be an infectious agent not only for the population of turtles but also for other species of animals inhabiting their natural environment. Moreover, the undesirable properties of water quality caused by the presence of drug-resistant aeromonads could have a negative impact on human health.

## 1. Introduction

The European pond turtle *Emys orbicularis* (L., 1758) is found in Eastern and Central Europe, Mediterranean countries, North Africa, and Northern Europe, including England, Denmark, and Sweden [1]. It is covered by the Bern Convention [2], protected by European Union laws, and listed in annexes II and IV of the Habitat Directive–Council Directive 92/43/EEC [3]. *E. orbicularis* is globally classified as Near Threatened on the IUCN Red List [4] and listed in the Polish Red Data Book of Animals [5]. *E. orbicularis* is an endangered species in Poland primarily due to such agrarian treatments as draining wetlands and the flood control of rivers, as well as the progressive urbanization of natural habitats. In Poland, *E. orbicularis* was under full legal protection since 1935, but these protection measures were insufficient [6], and so the pond turtle is currently under strict legal protection by the Regulation of the Ministry for the Environment [7].

The changing environmental conditions and progressive pollution of the natural habitats of these animals have a considerable impact on their health, which in turn promotes bacterial and fungal infections [8]. A particular problem may be the contamination of the environment with antibiotics, which in turn can affect the composition of the microbiota of the turtle digestive tract, increasing the proportion of species that can potentially cause both local and systemic infections [9]. The screening studies carried out so far in these animals have shown that the most important bacterial species in the microbiota of *E. orbicularis* include *Pseudomonas diminuta*, *Citrobacter freundii*, *Escherichia coli*, *Chryseobacterium*, *Enterobacter* sp., *Salmonella* sp., and *Aeromonas* sp. [8,10,11,12,13,14]. Among microorganisms that have the greatest potential to cause infection are those of the genus *Aeromonas* [15].

The representatives of the genus *Aeromonas* are Gram-negative, rod-shaped, facultative anaerobic, non-spore-forming bacteria that are widely distributed in water and are recognized as etiological agents for fish, amphibians, and reptiles, including turtles [10,13,15,16]. *Aeromonas* spp. are documented as a cause of dermatitis, stomatitis, rhinitis, pneumonia, osteomyelitis, and septicemia in captive green turtles (*Chelonia mydas*) [17,18], and skin diseases in captive loggerhead turtles (*Caretta caretta*) [19] and captive Kemp’s ridley turtles (*Lepidochelys kempii*) [20]. Moreover, *Aeromonas* play a significant role as opportunistic pathogens in humans, causing a variety of gastrointestinal infections, wound infections, diarrheal illness, septicemia, meningitis, cellulitis, ophthalmitis, endocarditis, and biliary tract infections [21,22]. The pathogenicity of the bacteria is complex and strictly connected with multiple virulence factors such as cytotonic heat-labile (alt) and cytotonic heat-stabile enterotoxins (ast), cytotoxic heat-labile enterotoxin (act), aerolysin (aer), flagellin (fla), elastase (ela), serine protease (ser), lipase (lip), collagenase (acg), DNase (exu), and cholesterolacyltransferase (gcaT) [23,24,25]. The presence of these virulence factors allows the bacteria to colonize, invade, and overcome the immune response mechanism of the host, resulting in an infection that generates the disease [25]. Therefore, *Aeromonas* species are considered to be emerging pathogens with increasing clinical significance in aquaculture and human health.

An additional factor increasing the potential infectivity and problems with combating *Aeromonas*-related infections is the increasing drug resistance of these bacteria. The antibiotic susceptibility of fish, human, and environmental isolates of *Aeromonas* spp. is extensively studied [26,27], but less is known about strains isolated from aquatic turtles, especially freshwater species [8].

Since aeromonads are commensally widespread in the intestines of wild and domesticated animals, they can also readily contaminate the environment, especially water sources, increasing the risk of infection with these microorganisms. Hence, this study was conducted to determine the resistance and virulence patterns of *Aeromonas* spp. originating from free-ranging European pond turtles and their environment.

## 2. Materials and Methods

### 2.1. Animal and Water Sample Collection

Our study included 74 adult European pond turtles (aged from 2 years to several decades) of both sexes from Polesie National Park (48°27′ N, 23°10′ E). The study was approved by the Local Ethics Committee for Experiments on Animals (resolution 42/2012 in accordance with DB/KKE/PL-60/2003). Cloacal swabs were collected during telemetry testing conducted in the park from May to July 2012. Water samples (*n* = 15, from 15 sampling points) from the turtle reservoirs were collected aseptically in sterilized glass bottles simultaneously. The samples were analyzed within 24 h, following transport in refrigerated containers at 4–8 °C.

### 2.2. Isolation and Species Identification

The swabs were initially incubated in 2 mL of buffered peptone water (24 h at 28 °C) and turbid growth cultures were streaked on selective Rimler-Shotts medium (RS) (Himedia). The water samples (100 µL per plate) were inoculated directly onto the RS medium. Single yellowish colonies were purified on the *Aeromonas* agar base (AAB) with ampicillin (2.5 mg/500 mL medium) (Oxoid). After incubation in aerobic conditions, the colonies (one colony from each positive plate) were isolated and identified on the basis of colony morphology, Gram staining, and biochemical characteristics. *Aeromonas* isolates were identified to the genus level using the following tests: cytochrome oxidase activity (strip OXItest, Erba Lachema, Brno, Czech Republic), growth on ABB, and sensitivity to 10 and 150 μg discs of the vibriostatic agent 0/129 on an agar base (Termofischer Scientific). Phenotypic biochemical properties were also tested with a commercial diagnostic kit NEFERMtest 24 (Erba Lachema, Brno, Czech Republic) according to the manufacturer’s instructions.

Further species identification was conducted with matrix-assisted laser desorption/ionization time-of-flight mass spectrometry (MALDI-TOF-MS), as previously described by Vávrová et al. [28]. Samples were prepared according to the standard extraction protocol [29]. Protein analysis by MALDI-TOF-MS was performed in an UltrafleXtreme mass spectrometry unit (Bruker Daltonik GmbH, Bremen, Germany). Prior to the analysis, the mass spectrometer was externally calibrated with a Bacterial Test Standard (Bruker, Germany) containing a previously prepared extract of *Escherichia coli* DH5 alpha. To assess the reproducibility of MALDI-TOF-MS identification, the analysis was repeated three times for each sample and bacterial spectra were acquired using flexControl 3.0 software. Then, they were analyzed using BrukerBiotyper 3.0.

The following score values proposed by the manufacturer were applied: a log(score) of ≥2 indicated good identification at the species level, a log(score) between 1.70 and 1.99 indicated a close relationship at the genus level, and log(score) of <1.7 indicated unreliable identification.

Proteomic identification was confirmed using the molecular method based on the restriction fragment length polymorphism (RFLP) of the 16S rRNA gene. The restriction enzymes *Alu*I and *Mbo*I or *Nar*I and *Hae*III (ThermoFisher Scientific, Waltham, MA, USA) were selected for the present study [30]. The following reference strains were used as a control: *A. hydrophila* CECT 839^T^, *A. veronii* CECT 4257, *A. salmonicida* CECT 894^T^, *A. bestiarum* CECT4247^T^, *A. allosaccharophila* CECT 4199^T^, *A. salmonicida* CECT 894^T^, and *A. bestiarum* CECT4247^T^. The molecular sizes of the fragments obtained were estimated using the Stand-Alone Gel Documentation Imaging Bio-1D++ (Vilber Lourmat, Collégien, France).

### 2.3. Antimicrobial Susceptibility Testing

The susceptibility of the isolated bacteria was evaluated with the microdilution method in accordance with the requirements of the Clinical and Laboratory Standards Institute M45-A2 [31]. The minimum inhibitory concentrations (MICs) of ampicillin, cefotaxime, ciprofloxacin, doxycycline, erythromycin, gentamicin, meropenem, sulfamethoxazole, and tetracycline antimicrobials (Sigma-Aldrich, Schnelldorf, Germany) were tested in the range concentrations of 0.06–64 µg/mL, with the exception of sulfamethoxazole (0.25–256 µg/mL). The reference strain, *E. coli* ATCC25922, was used as a quality control. The breakpoints recommended by CLSI [31] and EUCAST [32] were used; if not defined, the breakpoint values described by Stratev et al. [33] and Scarano et al. [34] were applied. The production of carbapenemases was confirmed using the modified carbapenem inactivation methods: mCIM and eCIM [35]

The MAR index of the isolates against the tested antibiotics was calculated based on the following formula [36]: MAR index = X/(Y × Z), where X–total of bacteria resistant to antibiotics, Y–total of antibiotics used in the study, and Z–total of isolates.

### 2.4. Detection of Virulence Genes

Total DNA was extracted from the identified *Aeromonas* isolates utilizing a Bacterial and Yeast Genomic DNA Purification Kit (Eurx, Gdańsk, Poland), according to the manufacturer’s guidelines. The isolates were tested for the presence of selected virulence genes (*aer*, *fla*, *lip*, *hly*A, *ast*, *alt*, *act,* and *ela*). The primers and cycling conditions used for the detection of virulence genes were as previously published (Table S1). All reactions were performed in a thermal cycler (T Personal thermal cycler–Biometra GmbH, Goettingen, Germany) using Gold Taq MIX (Syngen Biotech, Wrocław, Poland), appropriate primers (Genomed, Warsaw, Poland), and 1 μL of DNA template.

### 2.5. Statistical Analysis

Statistical analysis was performed in Statistic aver. 13.3 (Statsoft, Warsaw, Poland) and the level of significance was set at (*) *p* < 0.05 and (**) *p* < 0.005. The relationship between the sex of the turtle and the frequency of isolation of bacteria was carried out using the Χ^2^ test. The inference about the dependence of *Aeromonas* origin (animal or water) was carried out using the Student’s t-test. The assumptions of normality and homogeneity of variance were performed using the Shapiro–Wilk test and F test, respectively. The Student’s t-test was used in the case of determining the statistical significance of the biochemical properties used for microbial identification at the species level.

The degree of the variation frequency of the virulence genes of all *Aeromonas* isolates was tested by ANOVA. Then, the Student’s t-test was used for statistical evaluation of the frequency of the virulence genes between the representative species and between these species and all isolates. Statistical evaluation of virulence genes with the greatest importance in the analyzed bacterial species was performed using the post hoc Tukey HSD test.

## 3. Results

A total of 74 European pond turtles (*E. orbicularis*) of both sexes (41 female and 33 male adult turtles) and 15 water samples taken in Polesie National Park were included in the study (Eastern Poland). No exotic freshwater turtles were captured or observed in this area. All turtles appeared healthy; a few had missing digits, but no purulent nasal discharge or other breathing disorders were observed.

After culturing on the selective AAB medium, circular, concave, and blue-green colonies suspected of being *Aeromonas* (*n* = 42) were collected for further analysis (Table 1). All isolates were confirmed as oxidase-positive Gram-negative rods and classified into the *Aeromonas* genus according to the results obtained using the commercial diagnostic kit NEFERMtest 24. Regardless of the species, the results of nine tests (arginine dihydrolase, N-acetylglucosaminidase, acid production from mannitol, trehalose, galactose, maltose, and saccharose, gamma-glutamylo-transferase, as well as phosphatase) were positive for all the isolates. Moreover, all isolates were negative for the other seven tests: urea hydrolysis, ornithine decarboxylase, acetamide and malonate utilization, and acid production from D-xylose and inositol. The other eight of the twenty-four tests yielded variable results within a species for the same biochemical feature (Table 2).

The identification by MALDI-TOF-MS allowed the assignment of all the isolates to four different species: the largest population was *A. veronii* (*A. veronii* DSM 17,676 HAM, *n* = 21; *A. veronii* CECT 4257T DSM, *n* = 2; *A. veronii CECT* 4199T DSM, *n* = 1; *A. veronii* DSM 11576T HAM, *n* = 1) followed by *A. hydrophila* (*A. hydrophila* CECT 839T DSM, *n* = 10; *A. hydrophila* subsp. *hydrophila* DSM 30187T DSM, *n* = 3), *A. salmonicida* (*A. salmonicida* subsp *salmonicida* CECT 894T DSM, *n* = 3), and *A. bestiarum* (*A. bestiarum* CECT 4227T DSM, *n* = 1) (Table 3). According to the NCBI identifier (Bruker Daltonik MALDI Biotyper), most of the scientific names of *Aeromonas* species identified agreed with the species of the matched pattern with the exception of *A. veronii* CECT 4199T DSM and *A. veronii* DSM 11576T HAM, for which the scientific name according to the NCBI base was determined as *A. allosaccharophila* (Table 3). The level of identification for all the isolates was high: log (score) ≥2 (from 2.215 to 2.455) allowing for the reliable identification at the species level according to the manufacturer.

Based on the molecular identification, four different restriction profiles were demonstrated, which corresponded to five different species: *A. veronii*, *A. hydrophila*, *A. salmonicida* or *A. bestiarum*, and *A. allosaccharophila* (Table 3). Considering the scientific names of the matched patterns, the species identification determined with MALDI-TOF-MS and RFLP of 26S rRNA were fully confirmed in relation to the isolates tested.

The comparison of all methods used for identification showed that the compatibility of species identification was obtained at a reliable level between the commercial biochemical kit (NEFERMtest 24) and MALDI-TOF-MS/RFLP of 26S rRNA only for nine (21.4%) isolates of *A. hydrophila* and *A. salmonicida* (seven and two isolates, respectively) (Table 3).

Of the 74 turtles tested, 43.2% were found to be carriers of four different species of *Aeromonas* (Table 1). This group included 21 (28.4%) females and 11 (14.6%) males. There was no statistically significant difference between males and females with regard to the distribution of the percentage and species of bacteria. Of the 15 water samples, 10 (66.6%) were contaminated with a single species of *A. hydrophila*, *A. veronii*, or *A. bestiarum* (Table 1). It was not possible to determine at a statistically significant level whether the occurrence of *Aeromonas* was associated with adult turtles inhabiting water reservoirs, or whether the water itself was the source of the contamination.

The PCR amplification of the virulence genes from the template DNA of all 42 *Aeromonas* isolates from the European pond turtle was carried out. Oligonucleotide primers, specific for the toxin-encoding genes, allowed the amplification of an appropriate length of products (Table S1) for *aer*, *alt*, *ast*, and *act* in 76.2%, 64.3%, 59.5%, and 73.8% of the isolates, respectively (Table 4). A positive result for the amplification of the other genes, i.e., *fla, lip, hly*A, and *ela*, was obtained in 90.5%, 45.2%, 73.8%, and 71.4% of the strains, respectively (Table 4). The prevalence of individual virulence genes among the *Aeromonas* isolates was distributed as follows: the most frequent virulence gene, *fla*, was detected in 86.9% of *A. veronii* and 92.3% of *A. hydrophila* and all strains representing *A. salmonicida*, *A. allosaccharophila*, and *A. bestiarum*. The *Aer* gene was detected in 91.3% of *A. veronii*, 53.8% of *A. hydrophila*, all of *A. salmonicida*, and one of the two strains of *A. allosaccharophila*. The elastase-encoding gene was detected in all *A. salmonicida* and *A. bestiarum* strains, one strain of *A. allosaccharophila,* and in 65.9% and 76.9% of *A. veronii* and *A. hydrophila* strains, respectively. The *hly*A gene was detected in *A. veronii* (78.2%), *A. hydrophila* (84.6%), and single strains of *A. allosaccharophila* and *A. salmonicida*. Enterotoxin genes were found in different proportions; however, the *act* gene was not detected in *A. allosaccharophila* and *A. bestiarum*. The latter species did not contain the *alt* gene either (Table 4).

The results of the antimicrobial susceptibility of the isolates are shown in Table 5. All *Aeromonas* isolates were found to be resistant to ampicillin, while 62.0%, 40.5%, and 40.5% of the strains were resistant to sulfamethoxazole, colistin, and erythromycin, respectively. All strains were susceptible to chloramphenicol, gentamicin, doxycycline, meropenem, and cefotaxime. The mCIM and eCIM test results confirmed the production of metallo-β-lactamases in the strains tested.

The majority of the isolates (54.8%) had an MAR index of over 0.2, with a value of 0.24 for all *Aeromonas* isolates (Table S2). The MAR index ranged from 0.09 to 0.27 for the *A. veronii* strains, from 0.18 to 0.36 for the *A. hydrophila* strains, and from 0.09 to 0.18 for *A. allosaccharophila*. Its value for the *A. salmonicida* and *A. bestiarum* strains was 0.36.

## 4. Discussion

Due to the loss of suitable habitats, the European pond turtle, *E. orbicularis,* is regarded as endangered in Poland. At present, only a single large population (1500–2000 turtles) exists in eastern Poland (Leczna-Wlodawa Lake District) and there are only a few small populations in the rest of the country [6]. Under the Regulation of the Minister for the Environment on the Protection of Animal Species, *E. orbicularis* is under strict protection and requires active protection and designated areas of protection.

*Aeromonas* spp. bacteria are often isolated as an etiologic agent of diseases in ectothermic animals from healthy and diseased animals [13,14,37]. The wide distribution of *Aeromonas* spp. in the aquatic environment and aquatic turtles was previously reported [10], and our results support these findings. Wild turtles excrete a large amount of *Aeromonas* in their feces. Water contaminated by potentially pathogenic strains of aeromonads is a source of infection for humans and animals.

Due to the lack of a standardized effective and repeatable procedure, we used a multi-stage process for the isolation and identification of *Aeromonas* strains from stool samples of healthy animals and their living environment. The use of these types of analytical profile index tests based on the analysis of the biochemical profile generally allowed the reliable identification of the isolated strains, but only at the genus level [25,38]. Similar results were obtained by other authors [39], who showed a very low level of discrimination with regard to species identification within the genus of *Aeromonas*, using biochemical procedures. The low level of species discrimination based on biochemical properties mainly concerned environmental isolates due to their much greater diversity of species compared to the clinical isolates [25]. As shown in our research, it was not possible to determine the set of biochemical properties that ensured the reliable identification of the microorganism at the species level. Even by narrowing the inference to tests of the two most frequently isolated species (*A. hydrophila* and *A. veronii*) and taking into account the characteristics of the variables within a species, we did not determine a statistically significant level of biochemical properties as a diagnostic indicator. Therefore, the use of biochemical identification as the only diagnostic method does not seem to be a good solution.

Matrix-assisted laser desorption/ionization time of flight mass spectrometry (MALDI-TOF MS) is a relatively effective tool recently used in the routine identification of *Aeromonas* clinical isolates and, as shown by our research, in the consistency of identification between the results of molecular methods. In most cases, based on the protein profile, this is completed. The level of identification of the isolates was high in our study: a log (score) of >2.2 indicated good identification at the species level, according to the score values proposed by the manufacturer. As in the case of biochemical identification, the only problem was posed by closely related species [40,41] and the need to constantly enrich the comparative database with new spectra, which significantly increased the percentage of correct identifications [41].

Species identification is a very important factor that often determines diagnostic success. Nevertheless, in the case of the genus of *Aeromonas*, its taxonomy is still an open and debatable topic, as effectively shown by the example of *A. allosaccharophila*, which is still potentially regarded as *A. veronii* due to its high heterogeneity [42]. Currently, the genus of *Aeromonas* includes 36 species [25] and some species, including *A. veronii* and *A. hydrophila*, are the largest contributors to infections in both humans and animals. Therefore, the identification of these species is of the utmost importance in clinical diagnosis. As shown in our study, a combination of few different diagnostic procedures provides better results in the case of the identification of *Aeromonas*.

Previous studies showed that *Aeromonas* species isolated from water possessed various virulence factors and that these isolates could cause human diseases [43,44,45]. In aeromonads, disease was the result of a molecular symphony, with each virulence factor contributing to create a cumulative effect [46]. Cytotoxic enterotoxin (act), cytotonic enterotoxins (alt and ast), and aerolysin (aerA) played crucial roles in the establishment of infections and in causing diarrhea [46,47,48]. Castilho et al. [49] demonstrated that *Aeromonas* spp. could harbor and express virulence genes and reinforce the potential of *Aeromonas* as a human pathogen. An interesting aspect related to the research on the spread of virulence genes is the disclosure of the correlation between the species and a strictly defined set of virulence factors. However, the pathogenic nature of aeromonads is ambiguous. Nawaz et al. [50] described that 96.0% of *A. veronii* isolates from catfish harbored the aerolysin (*aer*A) gene and the cytotoxic enterotoxin (*act)* gene. Abu-Elala et al. [51] showed the presence of *act* and *aer* genes in all *A. veronii* biovar *sobria* isolates, whereas *act* and *lip* genes were present in all *A. hydrophila* isolates. In contrast, Pollard et al. [52] and Gonzalez-Serrano et al. [53] failed to detect the *aer* gene in any of the isolates. In our study, 76.2% of the aeromonad isolates from the European pond turtles harbored the *aer* gene, whereas 73.8%, 64.3%, and 59.5% of the isolates had the *act* gene, *alt* gene, and *ast* gene, respectively. However, as demonstrated by the presented study results, no statistically significant differences were found between the frequency of these genes in the two representative species, *A. hydrophila* and *A. veronii*, accounting for 85.7% of isolates, in which all of the tested virulence genes were present in 68.3% and 71.2%, respectively. Most likely, the virulence genes were not related to the *Aeromonas* species, and the set of virulence genes was determined by occupying the same ecological niche. Such an explanation is consistent with the mechanism of the widely described phenomenon of horizontal gene transfer (HGT), which occurs at a high frequency between species of the same genus. However, it should be emphasized that, in the present study, only two species constituted a representative pool; therefore, the statistical analysis could not reveal species-specific profiles. In a pool of several hundred of strains, Li et al. [48] showed a large variation in the profile of virulence genes depending on the *Aeromonas* species. In turn, such variability may potentially result in a somewhat different pathogenesis mechanism and clinical picture depending on the species that causes the infection.

The presence of virulence genes in both the strains isolated from infections and from clinically healthy animals or from the environment, does not allow an unambiguous conclusion regarding the zoonotic potential of a given strain depending on its virulence profile. For example, Wu et al. [45] found no direct association between the presence of the *aer*A, *hly*A, *alt*, and *ast* genes in *Aeromonas* isolates and the development of extraintestinal infections or bacteremia. Additionally, the research conducted by Chacon et al. [44] showed the presence of virulence genes in both clinical and environmental isolates at a similar level, as in the study carried out by Li et al. [48], who showed that, in both isolates from diarrheal patients and with water samples as sources of isolation, virulence genes appeared in various proportions. The only difference that was noted between the clinical and environmental isolates was the statistically higher frequency of aerolysin and enterotoxin-coding genes in the first group [44,48]. The presence of the other virulence genes in clinical, commensal, or environmental strains is much more comparable. Nevertheless, some authors indicate that the presence of specific combinations of a few genes, e.g., *hly*A and *aer*, are indicators of the possibility of inducing human diarrheal disease [54].

The production of extracellular enzymes such as lipase and elastase is an adaptation to the surrounding environmental conditions and the possibility of providing nutrients, thus characterizing environmental strains. On the other hand, these enzymes can act as virulence factors capable of damaging host tissues and increasing the possibility of invasion and can directly affect the host’s immune system by damaging leukocyte cell membranes or releasing free fatty acids [46,55]. Flagella, in turn, act as an adhesin, facilitating the colonization of the host’s mucous membranes in the first stage of infection and biofilm formation [46].

Nevertheless, the presence of a broad panel of virulence is not the only factor determining the pathogenesis of infection. In humans, the predisposing factors include coexisting immunocompromised disorders, diabetes mellitus, renal and cardiac problems, and the use of invasive medical procedures (colectomy, cholecystectomy, and elective surgery), which favor skin and soft tissue infection and even septicemia [56]. Similarly, when drinking water contains strains with genes encoding virulence factors such as cytotoxic enterotoxin, *act*/*hly*A/*aer*A, and cytotonic enterotoxins *alt* and *ast* become contributing factors to the development of human diarrheal disease, the likelihood of infection will certainly increase [57].

In animals, *Aeromonas* infections are also common, and numerous virulence factors are also demonstrated in the isolates in these cases. Nevertheless, the symptoms of *Aeromonas* infection develop most often in the presence of other factors, e.g., stress associated with captivity, the transport of animals, or an inappropriate ambient temperature [58].

In this study, all strains were resistant to ampicillin, which confirmed the validity of the results obtained in the preliminary study with the use of media supplemented with ampicillin. Most *Aeromonas* isolates had intrinsic or a chromosomally mediated resistance to ampicillin; therefore, this phenomenon was used for targeted isolation [59]. Similar results were observed by other researchers [26,60,61], who reported *Aeromonas* strains as resistant to ampicillin and penicillin. Moreover, *Aeromonas* produce β-lactamases such as cephalosporinase, penicillinase, and metallo-β-lactamases. These enzymes hydrolyze carbapenems such as meropenem [62]. In our study, 14.3% of the isolates were intermediately resistant to meropenem at the level of 8 µg/mL in all strains, with the highest number of strains (*n* = 5) belonging to the species *A. hydrophila*. The ability to produce metallo-β- lactamases was also confirmed using the mCIM and eCIM tests. Although, according to the CLSI standard, these tests were intended to detect carbapenemases in *Enterobacteriaceae* and *Pseudomonas aeruginosa*, studies have shown that they are also highly reliable in the detection of carbapenem resistance in *Aeromonas* spp [63,64]. Low rates of resistance to meropenem were also observed by Figueira et al. [65] and Aravena-Román et al. [66]. However, the *Aeromonas* strains isolated from seafood had a much higher resistance to these antimicrobials, even reaching 39–42%, with the most common resistance being to imipenem [67,68]. Due to the status of carbapenems in medicine as a group of drugs as a last resort, this very worrying trend should be monitored, since bacteria of the genus *Aeromonas* coould become donors of genes encoding metallo-β-lactamases for other Gram-negative species of bacteria.

This study differs from most other investigations in that sulfamethoxazole was tested alone rather than in combination with trimethoprim. Kämpfer et al. [69] tested clinical and nonclinical *Aeromonas* isolates against sulfamethoxazole and found 36% of them to be resistant, compared to 62% in our study. However, in the studies conducted by Scarano et al. [34], a 92.3% resistance level for sulfadiazine tested alone was found among strains isolated from mariculture farms.

The erythromycin and colistin resistance was at the same level of 40.5%. Only the *A. salmonicida, A. hydrophila*, and *A. bestiarum* isolates were resistant to colistin, which was consistent with the observation reported by Fosse et al. [70], who showed that resistance to colistin could be used as an additional criterion to differentiate the *A. hydrophila* complex from other susceptible species (e.g., *A. veronii*). In turn, in the case of erythromycin resistance, in addition to the erythromycin-resistant *A. salmonicida, A. bestiarum* (all resistant), and *A. hydrophila*, we noted erythromycin-resistant *A. veronii* strains (21.7%). Macrolide resistance was one of the more frequently reported types of insusceptibility among *Aeromonas* strains isolated from aquaculture [34] and, together with a resistance to ampicillin, sulfonamides, streptomycin, and trimethoprim, it formed the most commonly reported resistance profile in the *Aeromonas* genus [34].

The resistance profile obtained in the current study differs significantly from the *Aeromonas* susceptibility profiles isolated from aquaculture, including food-producing animals and ornamental fish, and the multi-drug resistance profile (MDR) is much more frequently reported in these groups due to the targeted antibiotic therapy [71]. For these groups, the resistance panel is much broader and is most often determined by the range of antimicrobials used, since drug resistance usually develops as a result of selective pressure [72].

The occurrence of MAR aeromonads in water and wildlife is recognized as an important public health hazard. The MAR index illustrates the spread of bacterial resistance in a given population. A value of the MAR index above 0.2 identifies bacteria isolated from sites with a higher risk of contamination where antibiotics are used frequently. A MAR index ≤ 0.2 identifies strains from an environment where antibiotics are used rarely or not at all [36]. To date, several studies reported the horizontal transfer of plasmids encoding MAR in *Aeromonas* species pathogenic for fish [73], but very little information is available on the MAR of bacterial pathogens of wild turtles in Poland. In our study, the MAR indices exceeded the 0.2 limit in 54.8% of isolates. This indicated that the isolates in this study originated from a source(s) at risk of microbial contamination. However, the resistance profile for the genus tested was predictable considering ampicillin and colistin resistance as chromosomally encoded resistance.

Polesie National Park, where the turtles live, is located in a region surrounded by areas used primarily for agriculture, including large-scale food-producing animal husbandry, which may be the cause of the appearance of resistance, including resistance genes and drug-resistant strains, as a result of the contamination of the environment with antibiotics used on farms. The most common contamination factor is surface fresh water, which easily distributes residual antibiotics in the environment [74]. The results of our previous research showed how serious the problem of resistance was. We observed a much higher percentage of multidrug-resistant indicator bacteria in other species of free-living animals, but the studied groups were characterized by either much greater synanthropization compared to the currently tested turtles or a very diversified diet (e.g., carnivorous mammals), allowing for an easier accumulation of drug-resistant microorganisms [75,76]

Fortunately, there are still several antibiotics to which *Aeromonas* are susceptible or almost completely susceptible to, including chloramphenicol, tetracycline, ciprofloxacin, gentamicin, doxycycline, and cefotaxime. However, our research has a limitation. Samples were taken a few years ago and resistance profiles may have changed over those years. Nevertheless, in the light of the currently planned studies in the same group of animals, the results obtained will constitute a reference point and will most likely show the potential dynamics for changes in antimicrobials resistance in the natural environment of turtles. Therefore, due to the high dynamics of the increase in drug resistance, which is observed worldwide, the periodic monitoring of drug susceptibility in pristine environment should be carried out.

## 5. Conclusions

This is the first study providing a general picture of the prevalence and presence of virulence-related genes, and the antimicrobial susceptibility profiles of *Aeromonas* species in free-living European pond turtles in Polesie National Park (Poland) and their environment. In the light of the mechanisms of activity of individual virulence factors and their high percentage of occurrence in the tested samples, the virulence genes detected in this study indicate the potential pathogenicity of the isolates, as well as the possible risk posed to human health. Moreover, our study confirms that free-living turtles can act as reservoirs of resistant *Aeromonas* species, despite the low impact of the anthropization of their environment (the National Park is under legal protection). This phenomenon highlights the excessive use of antibiotics in animal production, which promotes the increasing emergence of drug-resistant strains, even in potentially pristine environment or in animals that have never received targeted therapy.

## Figures and Tables

**Table 1:** Prevalence of *Aeromonas* species in turtles and water samples.

*Aeromonas* Species	Positive Samples		Total *n* = 89 (%)
	Animals *n* = 74 (%)	Water Samples *n* = 15 (%)	
*A. hydrophila*	8 (10.8)	5 (33.3)	13 (14.6)
*A. veronii*	19 (25.7)	4 (26.7)	23 (25.8)
*A. salmonicida*	3 (4.0)		3 (3.4)
*A. allosaccharophila*	2 (2.7)		2 (2.3)
*A. bestiarum*		1 (6.7)	1 (1.1)
Total, *n* (%)	32 (43.2)	10 (66.7)	42 (47.2)

**Table 2:** Differences in the biochemical profiles of *Aeromonas* species.

*Aeromonas* Species	Positive Biochemical Test Results							
	^1^ LYS	SCI	aGA	bGL	ARA	CEL	LAC	ESL
*A. hydrophila* (*n* = 13)	8	8	12			8	1	12
*A. veronii* (*n* = 23)	13	15	4	3		14		9
*A. salmonicida* (*n* = 3)		3		3				
*A. allosaccharophila* (*n* = 2)	1	1			1	2		
*A. bestiarum* (*n* = 1)		1						
Total (*n* = 42) (%)	22 (52.4)	28 (66.7)	16 (38.1)	6 (14.3)	1 (2.4)	24 (57.1)	1 (2.4)	21 (50.0)

^1^ LYS—lysine decarboxylase; SCI—citrate utilization; aGA—alpha-galactosidase, bGL—ß-glucosidase; acid from: ARA—L-arabinose; CEL—cellobiose; LAC—lactose; ESL—aesculin hydrolysis.

**Table 3:** Comparison of *Aeromonas* species identification results.

*Aeromonas* Species	Identification Methods		
	NEFERMtest 24	MALDI-TOF-MS	RFLP of 26S rRNA
*A. hydrophila* (*n* = 13)	*A. hydrophila* (*n* = 7) A. caviae (*n* = 4) A. sobria (*n* = 2)	*A. hydrophila* CECT 839^T^ DSM (*n* = 10) *A. hydrophila* subsp *hydrophila* DSM 30187^T^ DSM (*n*= 3)	*A. hydrophila* CECT 839^T^ (*n* = 13)
*A. veronii* (*n* = 23)	*A. caviae* (*n* = 12) A. sobria (*n* = 10) *A. ichtiosoma* (*n* = 1)	*A. veronii* DSM 17,676 HAM (*n* = 21) *A. veronii* CECT 4257^T^ DSM (*n* = 2)	*A. veronii* CECT 4257 (*n* = 23)
*A. salmonicida* (*n* = 3)	*A. salmonicida* (*n* = 2) *A. sobria* (*n* = 1)	*A. salmonicida* subsp *salmonicida* CEC^T^ 894T DSM (*n* = 3)	*A. salmonicida* CECT 894^T^/*A. bestiarum* CECT4247^T^ (*n* = 3)
*A. allosaccharophila* (*n* = 2)	*A. sobria* (*n* = 1) A. trota (*n* = 1)	*A. veronii* CECT 4199^T^ DSM (*n* = 1) A. veronii DSM 11576^T^ HAM (*n* = 1)	*A. allosaccharophila* CECT 4199^T^ (*n* = 2)
*A. bestiarum* (*n* = 1)	*A. caviae* (*n* = 1)	*A. bestiarum* CECT 4227^T^ DSM (*n* = 1)	*A. salmonicida* CECT 894^T^ and *A. bestiarum* CECT4247^T^ (*n* = 1)

**Table 4:** Prevalence of virulence genes in *Aeromonas* spp.

Virulence Genes	*A. hydrophila* *n* = 13	*A. bestiarum* *n* = 1	*A. salmonicida* *n* = 3	*A. veronii* *n* = 23	*A.* *allosaccharophila* *n* = 2	Total *n* = 42 (%)
*Aer* (aerolysin)	7		3	21	1	32 (76.2)
*Fla* (flagellin)	12	1	3	20	2	38 (90.5)
*Lip* (lipase)	9		2	8		19 (45.2)
*Hly*A (cytotoxin)	11		1	18	1	31 (73.8)
*Alt* (cytotonic enterotoxin)	6		2	17	2	27 (64.3)
*Ast* (cytotonic enterotoxin)	9	1	3	10	2	25 (59.5)
*Act* (cytotoxic enterotoxin)	7		2	22		31 (73.8)
*Ela* (elastase)	10	1	3	15	1	30 (71.4)

**Table 5:** Range of resistance to the antimicrobials tested.

*Aeromonas* Species	Number (%) of Resistance Strains					
	^1^AMP	CL	ERY	SSS	MER	CIP/TET
*A. hydrophila* (*n* = 13)	13 (100)	13 (100)	8 (61.5)	7 (53.8)	5 (38.5)	
*A. veronii* (*n* = 23)	23 (100)		5 (21.7)	15 (65.2)	2 (8.7)	1 (4.4)
*A. salmonicida* (*n* = 3)	3 (100)	3 (100)	3 (100)	3 (100)		
*A. allosaccharophila* (*n* = 2)	2 (100)				1 (50.0)	
*A. bestiarum* (*n* = 1)	1 (100)	1 (100)	1 (100)	1 (100)		
Total (*n* = 42)	42 (100)	17 (40.5)	17 (40.5)	26 (62.0)	8 (19.0)	1 (2.4)

^1^AMP—ampicillin (MIC breakpoint ≥ 8 µg/mL), CL—colistin (≥4 µg/mL), ERY—erythromycin (≥8 µg/mL), SSS—sulphamethoxazole (≥128 µg/mL), MER—meropenem (≥8 µg/mL), CIP—ciprofloxacin (≥2 µg/mL), TET (≥8 µg/mL).

## Data Availability

Data is contained within the article or supplementary material.

## Supplementary Materials

The following are available online at https://www.mdpi.com/article/10.3390/ani11102772/s1, Table S1: Sequence of oligonucleotides and PCR conditions used in the study, Table S2. Multiple Antibiotic Resistance Index of the *Aeromonas* spp. tested.
