# Supplementary material for: Virulence and Antimicrobial Resistance Pattern of Aeromonas spp. Colonizing European Pond Turtles Emys orbicularis and Their Natural Environment. First Study from Poland
Source: Animals (Basel). 2021 Sep 23;11(10):2772. doi: 10.3390/ani11102772 (PMC8532793; doi:10.3390/ani11102772)
Supplement: Supplementary file 1 [file animals-11-02772-s001.zip › animals-1376852-supplementary.pdf]

Table S1. Sequence of oligonucleotides and PCR conditions used in the study

Gene	Primer Sequence 5′-3	Length (bp)	Reference
Aer (aerolysin)	F CCTATGGCCTGAGCGAGAAG<br>R CCAGTTCCAGTCCCACCACT	431	[Igbinosa and Okoh, 2013]
Fla (flagellin)	F TCCAACCGTYTGACCTC<br>R GMYTGGTTGCGRATGGT	608	[Igbinosa and Okoh, 2013]
Lip (lipase)	F ATCTTCTCCGACTGGTTCGG<br>R CCGTGCCAGGACTGGGTCTT	382	[Sen and Rogers, 2004]
*Hly*A (cytotoxin)	F GGCCGGTGGCCCGAAGATACGGG<br>R GGCGGCGCCGGACGAGACGGG	597	[Igbinosa and Okoh, 2013]
Alt (cytotonic enterotoxin)	F TGACCCAGTCCTGGCACGGC<br>R GGTGATCGATCACCACCAGC	442	[Sen and Rogers, 2004]
Ast (cytotonic enterotoxin)	F TCTCCATGCTTCCCTTCCACT<br>R GTGTAGGGATTGAAGAAGCCG	331	[Sen and Rogers, 2004]
Act (cytotoxic enterotoxin)	F AGAAGGTGACCACCAAGAACA<br>R AACTGACATCGGCCTTGAACTC	232	[Sen and Rogers, 2004]
Ela (elastase)	F ACACGGTCAAGGAGATCAAC<br>R CGCTGGTGTTGGCCAGCAGG	513	[Sen and Rogers, 2004]

Table S2. Multiple Antibiotic Resistance Index of the *Aeromonas* spp. tested

Species/Strains	Multiple Antibiotic Resistance Index (% of MAR index over 0.2)
**A. *hydrophila* (*n* = 13)**	
Ah5	0.36
Ah13	0.36
Ah17	0.27
Ah18	0.36
Ah22	0.27
Ah23	0.27
Ah28	0.36
Ah33	0.27
Ah35	0.36
Ah36	0.36
Ah39	0.27
Ah40	0.45
Ah42	0.18
**A. *veronii* (*n* = 23)**	
Av1	0.09
Av2	0.18
Av3	0.27
Av7	0.09
Av8	0.09
Av9	0.27
Av10	0.18
Av11	0.18
Av12	0.09
Av14	0.09

Av15	0.09
Av16	0.18
Av21	0.27
Av24	0.18
Av25	0.18
Av26	0.18
Av27	0.18
Av29	0.27
Av30	0.27
Av32	0.27
Av34	0.18
Av38	0.18
Av41	0.27
***A. salmonicida* (*n* = 3)**	
As4	0.36
As6	0.36
As20	0.36
***A. allosaccharophila* (*n* = 2)**	
Aa19	0.09
Aa32	0.18
***A. bestiarum* (*n* = 1) 0.36 (100)**	
**Total (*n* = 42)**	**0.24 (54.8)**
